# An investigation for phylogenetic characterization of human Pancreatic cancer microbiome by 16SrDNA Sequencing and Bioinformatics techniques

**DOI:** 10.21203/rs.3.rs-4140368/v1

**Published:** 2024-03-25

**Authors:** Colby Hunter, Khadimou Dia, Julia Boykins, Karrington Perry, Narendra Banerjee, Jazmine Cuffee, Erik Armstrong, Gabrielle Morgan, Hirendra Nath Banerjee, Anasua Banerjee, Santanu Bhattacharya

**Affiliations:** Elizabeth City State University campus of The University of North Carolina; Elizabeth City State University campus of The University of North Carolina; Elizabeth City State University campus of The University of North Carolina; Elizabeth City State University campus of The University of North Carolina; Elizabeth City State University campus of The University of North Carolina; Elizabeth City State University campus of The University of North Carolina; Elizabeth City State University campus of The University of North Carolina; Elizabeth City State University campus of The University of North Carolina; Elizabeth City State University campus of The University of North Carolina; Boston University Chobanian & Avedisian School of Medicine; Mayo Clinic Hospital

**Keywords:** Microbiome, Pancreatic Cancer, 16SrRNA, Phylogeny

## Abstract

Pancreatic cancer is a significant public health concern, with increasing incidence rates and limited treatment options. Recent studies have highlighted the role of the human microbiome, particularly the gut microbiota, in the development and progression of this disease. Microbial dysbiosis, characterized by alterations in the composition and function of the gut microbiota, has been implicated in pancreatic carcinogenesis through mechanisms involving chronic inflammation, immune dysregulation, and metabolic disturbances. Researchers have identified specific microbial signatures associated with pancreatic cancer, offering potential biomarkers for early detection and prognostication. By leveraging advanced sequencing and bioinformatics tools, scientists have delineated differences in the gut microbiota between pancreatic cancer patients and healthy individuals, providing insights into disease pathogenesis and potential diagnostic strategies. Moreover, the microbiome holds promise as a therapeutic target in pancreatic cancer treatment. Interventions aimed at modulating the microbiome, such as probiotics, prebiotics, and fecal microbiota transplantation, have demonstrated potential in enhancing the efficacy of existing cancer therapies, including chemotherapy and immunotherapy. These approaches can influence immune responses, alter tumor microenvironments, and sensitize tumors to treatment, offering new avenues for improving patient outcomes and overcoming therapeutic resistance. Overall, understanding the complex interplay between the microbiome and pancreatic cancer is crucial for advancing our knowledge of disease mechanisms and identifying innovative therapeutic strategies. Here we report phylogenetic analysis of the 16S microbial sequences of the pancreatic cancer mice microbiome and corresponding age matched healthy mice microbiome. We successfully identified differentially abundance of microbiota in the pancreatic cancer.

## Introduction

Pancreatic cancer is the eighth most common cancer in women and the tenth most common cancer in men. In both men and women, the number of new cases of pancreatic cancer have gone up by around 1% each year since the late 1990s. Worldwide, an estimated 495,773 people were diagnosed with pancreatic cancer in 2020.It is estimated that 50,550 deaths (26,620 men and 23,930 women) from this disease will occur in the United States in 2024. It is the fourth leading cause of cancer death in both men and women.^1,2^

The human microbiome refers to the diverse community of microorganisms, including bacteria, viruses, and fungi, that inhabit various parts of the body, such as the gastrointestinal tract. The microbiome plays a crucial role in maintaining health, influencing the immune system, and participating in various metabolic processes.^3–5^

There is evidence to suggest that alterations in the gut microbiota may contribute to chronic inflammation, which is a known risk factor for the development of pancreatic cancer.^6–8^ Inflammatory processes in the pancreas can create a microenvironment conducive to the growth of cancer cells. Changes in the composition of the gut microbiome, known as microbial dysbiosis, have been associated with various diseases, including cancer.^9–15^ Studies have explored whether specific microbial profiles or imbalances in the gut microbiome could be linked to pancreatic cancer. The microbiome can influence the host’s immune system, in the context of cancer, including pancreatic cancer, an altered microbiome may affect the immune response to tumors. Researchers are investigating whether modulating the microbiome could enhance the effectiveness of cancer immunotherapy.^16–22^ The microbiome is involved in the metabolism of dietary components. Dietary factors have been linked to pancreatic cancer risk, and the microbiome may play a role in how the body processes and reacts to these dietary factors. Researchers are exploring whether manipulating the microbiome could be a potential avenue for therapeutic interventions in pancreatic cancer. This includes strategies such as probiotics, prebiotics, and fecal microbiota transplantation (FMT).^23–27^

Henceforth, it is important to explore the role of microbiome in pancreatic cancer for investigating etiology, diagnostic, prognostic, and therapeutic intervention of pancreatic cancer.

We report in this manuscript identification of bacterial strains that are present in higher numbers in pancreatic cancer model mice fecal microbiome and bacteria’s that are in low numbers or absent in comparison to the fecal microbiome of healthy mice cohort. This study was done by 16SrDNA phylogenetic study by applying Next Generation Sequencing and Bioinformatics tools for identifying the microbial organisms. Thus, in this study, we report for the first time the human pancreatic cancer microbiota derived from a pancreatic cancer cell line originally developed from Pancreatic cancer tissue of a 51-year-old white male patient and created a xenograft mouse model, results of the study is as described in this manuscript.

## Materials and Methods

### Cell culture:

Human pancreatic cancer cell line, Panc-1 (ATCC CRL 1469) were purchased from American Type Culture Collection (ATCC; Manassas VA) and used with no further validations. For cell culture modified Dulbecco’s Eagle Medium (DMEM) were purchased from Gibco (Billings MT) and were supplemented with 10% Heat-Inactivated Fetal Bovine Serum (HI-FBS) (purchased from Gemini Bio-Products, Sacramento CA) and 1% Penicillin Streptomycin purchased from Gibco. Cells were cultured in a 150-mm dish till 80% confluency at 37°C in a humidified incubator maintained at 5% CO_2_ atmosphere.

### Animal protocol:

Female NOD-SCID-Gamma (NSG) mice aged 6–8 weeks were purchased from NCI and were housed in the institutional animal facilities on a 12:12 h light and dark cycle with 5 mice per cage. All animal procedures were approved by the Animal Care and Ethics Committee at Mayo Clinic. Approximately 1 × 10^6 luciferase transfected PANC-1 cells suspended in 100 uL of 1:1 PBS and matrigel and were inoculated orthotopically into the head of the pancreas of the mouse. The growth of the tumor was monitored at 7 weeks of tumor cell implantation using IVIS bio-imager (data not shown) after injecting Luciferin. Fecal materials were collected from the cage containing 5 tumor bearing mice. For control, fecal material was collected from age matched mice.

### Genomic DNA Extraction:

Fecal material was collected from Pancreatic cancer model mice cohort and healthy mice cohort at Mayo Clinic Jacksonville, FL. Genomic DNA was isolated from the cancer and control fecal material according to the manufacturer’s instructions using the ZymoBIOMICS^®^-96 MagBead DNA Kit (Zymo Research, Irvine, CA). The DNA was run on a 1% agarose gel to confirm the successful extraction and concentration measured using a Nanodrop instrument (VWR, USA).

### Control Samples:

The ZymoBIOMICS^®^ Microbial Community Standard (Zymo Research, Irvine, CA) was used as a positive control for each DNA extraction. The ZymoBIOMICS^®^ Microbial Community DNA Standard (Zymo Research, Irvine, CA) was used as a positive control for each targeted library preparation. Negative controls (i.e. blank extraction control, blank library preparation control) were included to assess the level of bioburden carried by the wet-lab process.

### Targeted Library Preparation:

The DNA samples were prepared for targeted sequencing with the Quick-16S^™^ Plus NGS Library Prep Kit (Zymo Research, Irvine, CA). These primers were custom designed by Zymo Research to provide the best coverage of the 16S gene while maintaining high sensitivity. Quick-16S^™^ Primer Set V3-V4 (Zymo Research, Irvine, CA) was used to amplify the targeted region of the microbial 16Sgene. The sequencing library was prepared using an innovative library preparation process in which PCR reactions were performed in real-time PCR machines to control cycles and therefore, limit PCR chimera formation. The final PCR products were quantified with qPCR fluorescence readings and pooled together based on equal molarity. The final pooled library was cleaned up with the Select-a-Size DNA Clean & Concentrator^™^(Zymo Research, Irvine, CA), then quantified with TapeStation^®^ (Agilent Technologies, Santa Clara, CA) and Qubit^®^ (Thermo Fisher Scientific, Waltham, WA).

### Sequencing:

The final library was sequenced on Illumina^®^ NextSeq 2000^™^ with a p1 (cat 20075294) reagent kit (600 cycles). The sequencing was performed with 30% PhiX spike-in.

### Absolute Abundance Quantification:

A quantitative real-time PCR was set up with a standard curve. The standard curve was made with plasmid DNA containing one copy of the 16S gene and one copy of the fungal ITS2 region prepared in 10-fold serial dilutions. The primers used were the same as those used in Targeted Library Preparation. The equation generated by the plasmid DNA standard curve was used to calculate the number of gene copies in the reaction for each sample. The PCR input volume (2 μL) was used to calculate the number of gene copies per microliter in each DNA sample. The number of genome copies per microliter DNA sample was calculated by dividing the gene copy number by an assumed number of gene copies per genome. The value used for 16S copies per genome is 4. The value used for its copies per genome is 200. The amount of DNA per microliter DNA sample was calculated using an assumed genome size of 4.64 × 106 bp, the genome size of Escherichia coli, for 16S samples, or an assumed genome size of 1.20 × 107 bp, the genome size of Saccharomyces cerevisiae, for ITS samples. This calculation is shown below:

Calculated Total DNA = Calculated Total Genome Copies × Assumed Genome Size (4.64 × 106 bp) × Average Molecular Weight of a DNA bp (660 g/mole/bp) ÷ Avogadro’s Number (6.022 × 1023/mole).

Bioinformatics Analysis: Unique amplicon sequences were inferred from raw reads using the Dada2 pipeline. Chimeric sequences were also removed with the Dada2 pipeline. Taxonomy assignment was performed using Uclust from Qiime v.1.9.1. Taxonomy was assigned with the Zymo Research Database, a 16S database that is internally designed and curated, as reference. Composition visualization, alpha-diversity, and beta-diversity analyses were performed with Qiime v.1.9.1. If applicable, taxonomy that have significant abundance among different groups were identified by LEfSe using default settings. Other analyses such as heatmaps, Taxa2SV_deomposer, and PCoA plots were performed with internal scripts.

## Result

**Figure 1:**
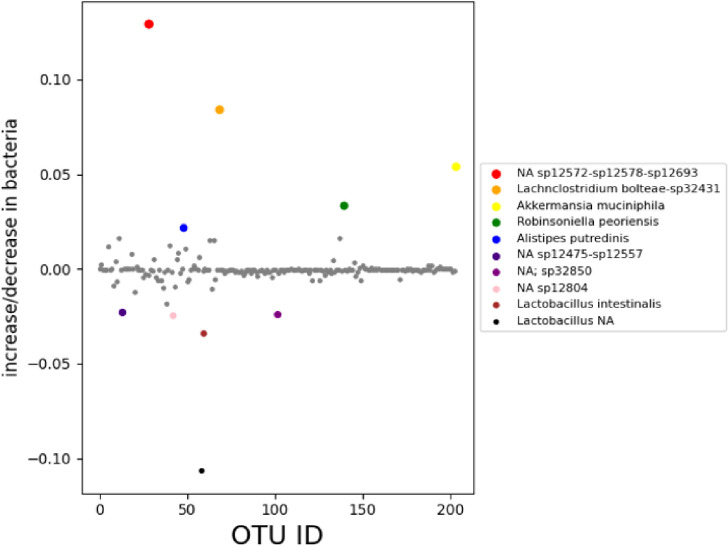
Differential bacterial population in pancreatic cancer xenograft mice model compared to age matched control mice. The x-axis represents the OTU ID for each bacterial species, while the y-axis represents the corresponding differences between the control group and cancerous group for each bacterial species.Positive and negative outliers are identified and shown using this method. The first positive outlier (red) shows the bacterium *NA sp12572-sp12578-sp12693*, the second positive outlier (orange) shows the bacterium *Lachnoclostridium bolteae-sp32431*, and the third positive outlier (yellow) shows the bacterium *Akkermansia muciniphila*. The fourth positive outlier (green) shows the bacterium *Robinsoniella peoriensis*, and the fifth positive outlier (blue) shows the bacterium *Alistipes putredinis*. The first negative outlier (purple) shows the bacterium *NA sp12475-sp12557*. The second negative outlier (magenta) shows the bacterium*NA sp32850*, the third negative outlier (pink) shows the bacterium *NA sp12804*, the fourth negative outlier (brown) shows the bacterium *Lactobacillus intestinalis*, and the fifth negative outlier (black) shows the bacterium *Lactobacillus NA*.

Phylogenetic analysis of the 16S microbial sequences of the pancreatic cancer mice microbiome showed overabundance of certain bacteria while absence of several strains when compared to healthy mice fecal microbiota, we present our data obtained in Table 1 and 2 and Figure 1 showing the relative abundance of different bacteria population in the pancreatic cancer and control mice. Several bacteria have been recognized as positive and negative outliers in our analysis confirming elevated or lowered presence in pancreatic cancer bearing mice compared to age matched controls respectively. Among these, *NA sp12572-sp12578-sp12693, Lachnoclostridium bolteae-sp32431, Akkermansia muciniphila,Robinsoniella peoriensis and Alistipes putredinis* presented by red, orange, yellow, green and blue colors respectively showed predominant presence in pancreatic cancer bearing mice (Figure 1). On the contrary, *NA sp12475-sp12557, NA sp32850, NA sp12804, Lactobacillus intestinalis*, and *Lactobacillus NA* were more abundant in control mice than pancreatic cancer bearing mice.

## Discussion

Our investigation in identifying differences in microbiota in human pancreatic model mice cohort feces in comparison to control mice cohort showed several unique bacterial strains that were present in abundance or absent in the fecal microbiome of pancreatic cancer bearing mice. Literature review informed the importance of varying abundance of microbiota in cancer and select bacteria are discussed below.

*Lachnoclostridium* bolteae was previously named *Clostridium bolteae*. This microbe is a gram-positive rod and an obligate anaerobe. It is a spore forming organism that resides within the human gut. *C. bolteae* has flagella which helps allow it to be an opportunistic pathogen. This, as well as several genes providing resistance to ampicillin, erythromycin, lincomycin, ciprofloxacin, and doxycycline make it a problematic constituent of the human microbiome.^28^ It has been linked to autism as well as pancreatic cancer in human beings.^29,30^

*R. peoriensis* is a gram-positive, spore forming, anaerobic bacillus that was characterized in 2003 from a swine manure sample and is an emerging human pathogen.^31^ It caused bacteremia in a patient with pancreatic cancer. Upon treatment with intravenous metronidazole the patient died of multiple organ failure within a month of being admitted to the hospital.^32^ In 2022, eight months after a prematurely terminated surgery, a Canadian woman with jejunal adenocarcinoma and endometrial cancer was admitted to hospital and analysis of blood and uterine samples revealed a *Clostridium perfringens* infection.^33^ A course of piperacillin-tazobactam, tobramycin and doxycycline was initiated for four days with resolution. Next, she was put on oral amoxicillin-clavulanate for five weeks. Five months later she came to the ER with several severe symptoms and blood samples were positive for *R. peoriensis* and *Clostridium difficile*.

*Alistipes putredinis* is a gram-negative, anaerobic bacterium that is commonly found in the human gastrointestinal tract. Colorectal cancer patients have been demonstrated to be enriched with *A. putredinis*.^34^ In IL-10 knockout mice (that also did not make the antimicrobial Lcn2 that prevents bacterial iron siderophores from functioning), *Alistipes spp*. proliferates in the right side of the murine colon (proximal, cecum) and promote polyp formation. *A. putredinis* has also been detected in nipple aspirate fluid in women with a history of breast cancer compared to healthy controls.^35^ Surprisingly, this organism is considered a favorable bacterium for the host when it comes to immunotherapy. Its presence in the gut is associated with successful Anti-PD-L1 therapy.^36^

*Akkermansia muciniphila* is a gram-negative, anaerobic organism. *A muciniphila* is a normal constituent of the mammalian gut microbiome. It is not motile, nor does it form spores. It enjoys the attention of microbiome researchers because it contributes to the turnover of intestinal mucous, tightens gut epithelial cell junctions, and stimulates the immune system. These capabilities make it an attractive probiotic candidate and viable alternatives to *Lactobacillus* spp. and *Bifidobacteria* spp.

There are dozens of studies providing evidence that this organism and its products contribute to positive health outcomes, including cancer. Luo et al demonstrated that extracellular vesicles (EV) derived from A. muciniphila could limit tumor size in the murine prostate cancer model.^37^ This effect may be due to the increase in GZMB+ and IFN-γ+ lymphocytes in tumors. Furthermore, the researchers observed that EV treated mice had more M1 macrophage in the tumor microenvironment. In addition, when THP-1 macrophages were exposed to EVs in vitro they assumed shape alterations and transcription profiles of the M-1 phenotype which potentiates neutralization of tumor cells.

Shi et al obtained tumor samples from patients with colorectal cancer.^38^ These ex vivo samples were processed and then challenged with *A. muciniphila* and IL-2. Apoptosis of tumor cells, the ratio of CD8+/CD4+ in CD3+ cells, CD80+CD86 in DD11c+ cells, and IFN-γ+ in CD3+ all were significantly higher than challenge with IL-2 or the microorganism alone. The melanoma and CRC murine models were consequently employed to test the effect of *A. muciniphila* and IL-2 on tumor volume and survival rate. *A. muciniphila* and IL-2 treated mice demonstrated statistically significant tumor volume and survival rate.

*A. muciniphila* has also shown synergy with cisplatin against lung cancer. Investigating the impact of *A. muciniphila* on Lewis Lung Cancer in mice, Chen et al found that mice treated with cisplatin and *A. muciniphila* had lower expression of ki67/GAPDH and p53/GAPDH at statistically significant levels compared to mice administered cisplatin only.^39^ Serum concentration of TNF-α, IL-6, and IFN-γ were also found to be significantly lower in *A. muciniphila* /cisplatin mice versus mice administered only cisplatin.

In a multi-institutional study, researchers found that in patients with NSCLC, RCC, or urothelial carcinoma being treated with d PD-1/PD-L1 mAb had significantly different outcomes based on if they had also received antibiotics proximal to the first cancer treatment.^40^ Progression free survival and overall survival were lower in those patients who took a course of antibiotics compared to those who did not. Next, the team found that metagenomic diversity corresponded with clinical outcomes in RCC and NSCLC patients that were newly diagnosed. Upon commencing PD-1 blockade therapy, the patients could be divided into those that responded to therapy and those that were non respondent. *A. muciniphila* was one bacterium that was at elevated abundance in the responders. Furthermore, germ free and specific pathogen free MCA-205 sarcoma mice given fecal microbiome transplants from responder NSCLC and RCC patients displayed smaller tumor sizes with PD-1 blockade than mice receiving non responder FMT or responder FMT without PD-1 blockade. To link a specific gut microbe to synergy with PD-1 blockade, gut dysbiosis was promoted in RET-melanoma mice. Then oral gavage with *A. muciniphila* alone or with *E. hirae* was performed to allow colonization of the gut. Tumor size was lower in mice receiving bacterial gavage relative to control but lowest in mice receiving both bacteria.

A more recent study by the previously mentioned group investigated the effectiveness of PD-1 blockade based on detection of *A. muciniphila* (Akk) in feces.^41^ The Akk^+^ cohort showed a 28% objective response rate compared to 18% in the Akk^−^ cohort. In Akk^+^ and Akk^−^ patients receiving solely PD-1 blockade, the ORR was 41% and 19% respectively. Transcriptomic analysis of tumor biopsies demonstrated distinct signatures between responders and non-responders to PD-1 blockade. These genes were relevant to activation of CD4+ T helper cells, exhaustion, and interferons.

This group also discovered that Akk^+^ patients had significantly different abundances of the bacterium. Fecal microbiome proportion ranged from 0.035% to 66.210%. This allowed the establishment of the Akk^high^ group (>4.799%) and an Akk^low^ group. Overall survival percentage was significantly higher in the Akk^low^ group (27.2 months) compared to Akk-(15.5 months), and Akk^high^ (7.8 months). In those patients receiving antibiotics, the overall survival percentage of Akk^low^ patients was more pronounced (27.2 months) compared to Akk^−^(15.5 months) and Akk^high^ (7.8 months). Akk^high^ also showed elevated levels of Lachnoclostridium bolteae after antibiotic exposure.

Interestingly, there are also other pockets of research that show a negative side to *A. muciniphila* especially in the murine colorectal cancer model.^42–44^ This could be due to subtle variations in experimental design such as the use of live/pasteurized forms of the bacteria, antibiotic usage, presence of dysbiosis, dosage, or route of administration.^45^

As its name implies, *A. muciniphila* is a chemoorganoheterotroph that utilizes every sugar found in mucin for carbon and energy.^46^ Some cancers are known to result in high levels of mucous secretion (e.g., adenocarcinomas, colorectal, gastric, lung, ovarian, and pancreatic). Thus, the elevated levels of *A. muciniphila* in the experimental group of our study could be due to the high amounts of mucous in the gastrointestinal tracts of these mice, which selected for the bacterium. This possibility invokes the concept of “driver” versus “passenger” bacteria in oncology studies.^47^ Oncobionts such as *Fusobacterium nucleatum* can become enriched in certain tissue where they interact with host cells to promote cancer. Once sufficient momentum has been attained, other bacteria become selected for and proliferate at these sites. Knowledge of bacteria physiology and metabolism must be leveraged in all future oncology studies to form a more accurate model of cancer. Fortunately, more sensitive biotechnological tools as well as the rise of integrated STEM research, and multidisciplinary teams are facilitating this course of action.

Our investigation in analysis of the pancreatic cancer model mice fecal microbiome by 16S rDNA sequencing and phylogenetic analysis resulted in identification of several bacterial strains that were either present in abundance or absent in the feces of cancer microbiome in comparison to the healthy mice. Further studies will be needed for clarifying the significance of these findings and the role of these bacteria in pancreatic cancer.
